# A Case of Thyroid Storm With Fatal Outcome due to Liver Failure

**DOI:** 10.1155/crip/1962799

**Published:** 2025-12-30

**Authors:** Kanako Suzuki, Satomi Makiuchi, Yukiko Kishida

**Affiliations:** ^1^ Department of Pathological Physiology, Faculty of Medicine, Masaryk University, Brno, Czech Republic, muni.cz; ^2^ Department of Pathology, Tokyo Teishin Hospital, Tokyo, Japan

**Keywords:** acute liver failure, autopsy report, centrilobular necrosis, fatal, hyperthyroidism, icterus, subacute hepatic necrosis, tachycardia, thyroid crisis, thyroid storm

## Abstract

We conducted an autopsy on a patient who succumbed to thyroid storm complicated by liver failure. As autopsies of thyroid storm are rare, we present the findings of the autopsy. A 70‐year‐old female patient who developed rapid onset acute liver failure passed away on the 15th day of hospitalization due to a thyroid storm. As in previously reported cases, lobular central necrosis of the liver was a predominant feature, and fibrosis was also seen. In this case, acute cardiac failure seemed to play a significant role as a cause of the liver failure. It may be crucial to prevent heart failure (HF), leading to hepatic ischemia before liver fibrosis develops.

## 1. Introduction

Thyroid storm, also known as thyrotoxic crisis, is considered an endocrine emergency due to its significant morbidity and mortality rates.

Although rare, the mortality rates associated with thyroid storm can be as high as 10%–20%. The clinical presentation is characterized by fever, tachycardia or supraventricular arrhythmias, and central nervous system manifestations like severe agitation, as well as gastrointestinal symptoms [1]. Liver function tests (LFTs) often show abnormalities in patients with thyroid storm, but acute liver failure (ALF) occurs rarely.

ALF is typically defined as the simultaneous development of encephalopathy and coagulopathy, and it occurs in the absence of any pre‐existing liver disease. Furthermore, it is characterized by an illness duration of fewer than 26 weeks [2].

In this article, we present a case involving a patient with a history of untreated Basedow’s disease who developed ALF and subsequently died as a complication of thyroid storm.

## 2. Clinical Summary

A 70‐year‐old female presented with jaundice, decreased appetite, and weight loss. Abnormalities in thyroid hormone levels were identified, leading to her transfer to our hospital for further evaluation and management.

There was no history of medical checkups for about 3 years, no previous diagnosis of Basedow’s disease, nor any family history of thyroid and/or autoimmune disease. Two days before hospitalization, a CT scan and abdominal ultrasound did not reveal any abnormalities in liver shape or size.

Upon admission, blood tests revealed elevated levels of FT3 and FT4, and the patient exhibited gastrointestinal symptoms such as diarrhea, jaundice, and liver dysfunction with elevated levels of transaminase (AST 127 IU/L and ALT 61 IU/L). While a definitive diagnosis of thyroid storm was made based on the Japan Thyroid Association (JTA) criteria, no organic change that would account for the raised bilirubin (bil) level, for instance, intrahepatic bile duct dilatation or cholangitis, was seen. The results of screening tests for autoimmune hepatitis and primary biliary cirrhosis were negative. Additionally, supplements and drug‐induced factors were ruled out, and it was concluded that the liver injury was due to thyrotoxicosis.

The patient experienced tachycardia and symptoms of HF associated with atrial fibrillation (AF), which was considered a possible symptom of a thyroid storm. Regarding AF, because of the lack of prior physical checkups, the etiologic history was not definitive, but based on the admission data, there was no prior awareness of palpitations. Furthermore, there were no signs of edema or pulmonary congestion at admission, suggesting that pre‐existing AF was unlikely. The overall clinical presentation did not meet the JTA diagnostic criteria for thyroid storm‐induced cardiac failure [3].

Burch–Wartofsky Point Scale (BWPS) score was 65 points; the estimated score is as follows: BT 37.6°C (5 points), gastrointestinal and liver dysfunction (10 and 20 points, respectively), and tachycardia in the cardiovascular system (10 points), totaling 45 points. Additionally, if the observed disorientation and drowsiness at admission were CNS symptoms, an extra 20 points would bring the total score to 65, supporting the diagnosis of thyroid storm [4]. On physical examination, the patient presented with tachycardia, diffuse thyroid enlargement, and hand tremors.

Ultrasound of the thyroid revealed diffuse increased blood flow, confirming the diagnosis of Basedow’s disease. The triggering factor for thyroid storm was unclear, as no evidence of infection was found.

Following treatment guidelines, the patient was started on methimazole (MMI) 30 mg/day, potassium iodide (KI) 200 mg/day orally, and hydrocortisone 300 mg/day. The treatment regimen underwent changes in the following course (Figure 1). On Day 4, plasma exchange and continuous hemodialysis and filtration (CHDF) were started daily, but the data on Day 6 showed FT3 5.66 ng/dL and FT4 15.2 ng/dL. After skipping plasma exchange on Day 7, blood on Day 8 showed FT3 7.10 ng/dL, FT4 19.1 ng/dL, and TRAb 82.0 IU/L, and another plasma exchange was performed. Determining that treatment so far was ineffectual, total thyroidectomy was considered. However, the patient was elderly (70 years old) and presented with ALF, decompensated HF with AF, and DIC. These conditions represented major perioperative risks, including coagulopathy, hemodynamic instability, and severe anesthetic risks, making immediate surgery impractical in this setting. Therefore, conservative management with intensive medical therapy was pursued instead.

Figure 1Clinical course and laboratory data during hospitalization. (a) The timeline of administered medications and TPE + CHDF sessions (▼). (b) The changes in T‐Bil, FT3, FT4, TRAb, AST, and ALT levels. The onset of hepatic encephalopathy, BNP elevation, and death (†) is indicated.

**Figure:**
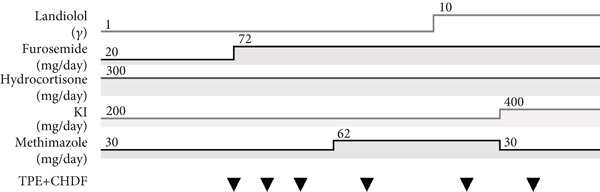
(a)

**Figure:**
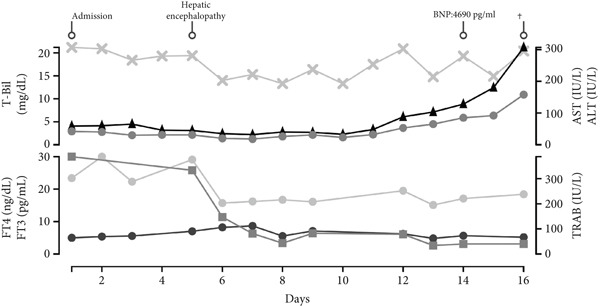
(b)

**Figure 2:**
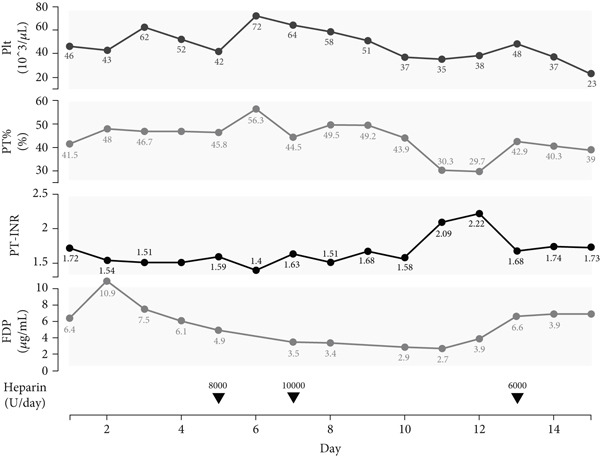
Clinical course of coagulation parameters and heparin therapy. Trends in platelet count (Plt, ×10^3^/*μ*L), prothrombin time activity (PT, percent sign), prothrombin time–international normalized ratio (PT‐INR), and fibrin/fibrinogen degradation products (FDP, micrograms per milliliter) are shown from Day 1 to Day 15. Arrows indicate the daily dose of unfractionated heparin (U/day) administered during the clinical course.

Despite frequent coagulation factor replacement and plasma exchange, there was no improvement in the prothrombin time activity percentage (PT%), suggesting a significant decrease in liver synthetic function (Figure 2). Moreover, with markedly elevated bil levels and minimal increase in AST/ALT, it was suspected to be an end‐stage liver failure with an acute course.

Due to the patient’s age and thyrotoxicosis, liver transplantation was not performed.

The management of acute HF was also challenging, and the patient’s general condition gradually deteriorated, resulting in her demise 15 days after admission. An autopsy was performed on the same day.

## 3. Pathological Findings

At autopsy, the patient’s height was 150 cm, her weight was 40.0 kg, and her skin and conjunctiva were visibly jaundiced.

Gross findings revealed that the thyroid gland weighed 102 g and showed diffuse enlargement with variable‐sized cystic structures. Histology showed marked proliferation of follicular epithelial cells without evidence of lymphocytic thyroiditis (Figures 3a and 4a).

Figure 3Macroscopic picture of thyroid, liver, and heart. (a) Thyroid weight 102 g, width 10 cm. (b) Atrophic with severe biliary stasis. (c) Left ventricular hypertrophy and fibrotic foci and right ventricular dilatation.

**Figure:**
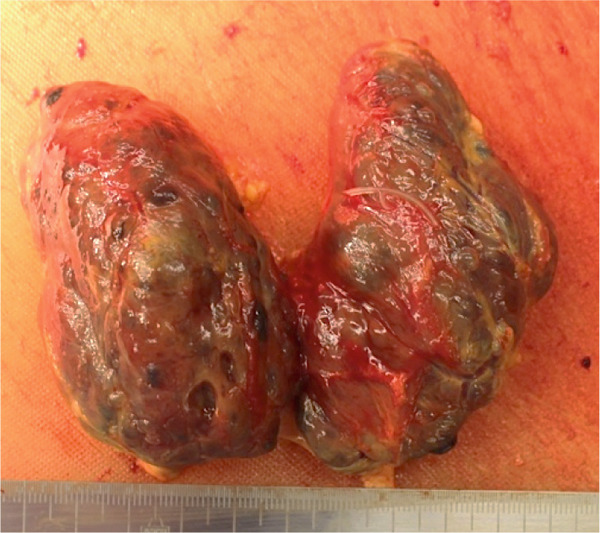
(a)

**Figure:**
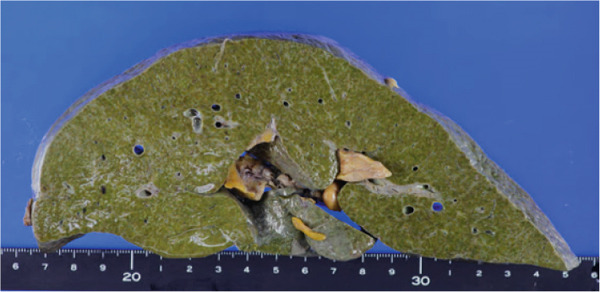
(b)

**Figure:**
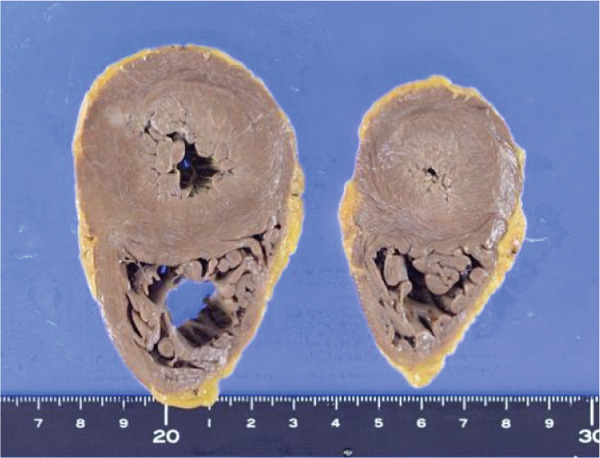
(c)

Figure 4Microscopic picture of the thyroid, liver, heart, and kidneys. (a) Follicular cell proliferation with irregularly shaped follicles and cysts. (b) Fibrotic enlargement of the portal area with pseudobile duct formation and lymphocytic inflammation. (c) Severe congestion, hepatocyte loss, and macro‐ and microvesicular fatty change. (d) Lobular shrinkage, widening of the portal area with bridging fibrosis (EMG staining). (e) Myocardial fibrosis and hypertrophy (EMG staining). (f) Herniation of the tubular epithelium.

**Figure:**
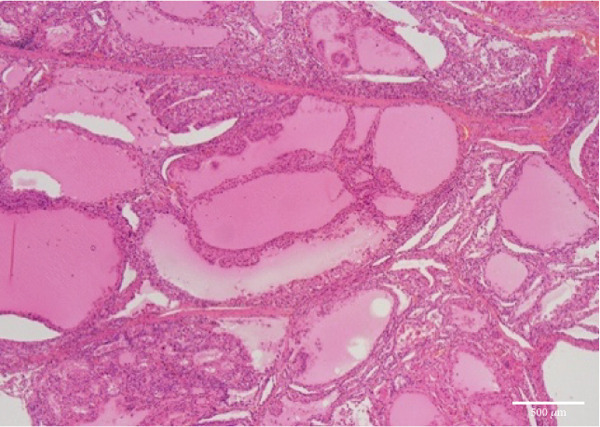
(a)

**Figure:**
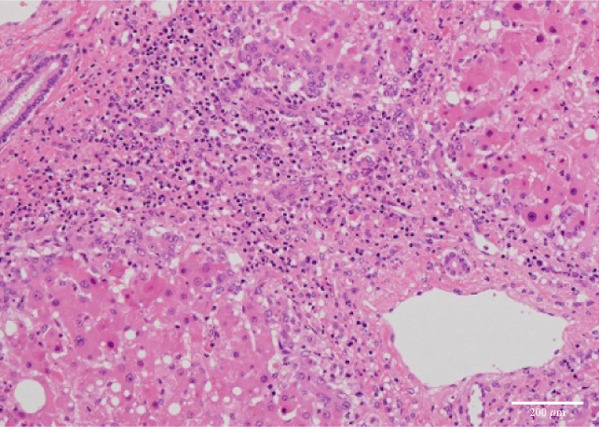
(b)

**Figure:**
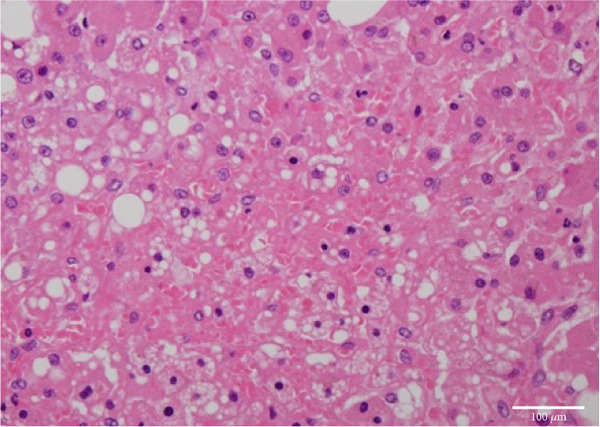
(c)

**Figure:**
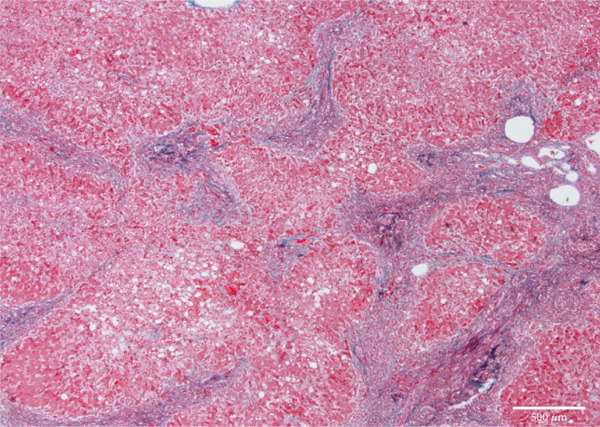
(d)

**Figure:**
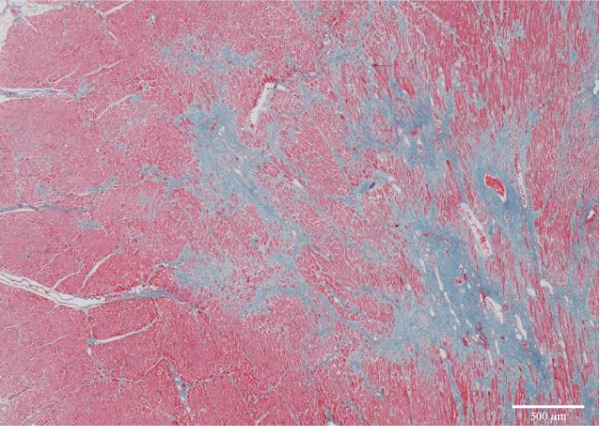
(e)

**Figure:**
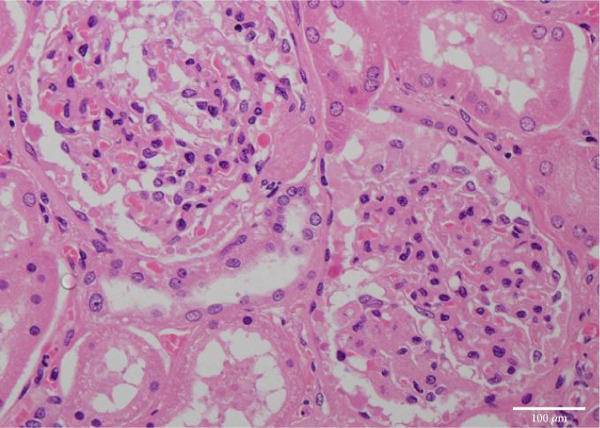
(f)

The liver weighed 700 g and exhibited fibrotic atrophy accompanied by severe jaundice (Figure 3b). Histological examination of liver tissue showed severe congestion in the sinusoids, cholestasis in hepatocytes and sinusoids, loss of hepatocytes, fibrotic enlargement of the portal area with pseudobiliary duct proliferation, early bridging fibrosis, lymphocytic infiltration, and mild macrovesicular and microvesicular steatosis. While there was some reactive neutrophilic infiltration, cholangitis was excluded, and no central venous occlusion was seen (Figures 4b, 4c, and 4d).

Heart weight was 360 g, with no significant increase in weight. The intracardiac blood volume was 600 mL, indicative of HF. Left and right ventricular wall thickness was 1.5 and 0.3 cm. Although there were no prominent atherosclerotic changes in the coronary arteries, the myocardium was hypertrophic with areas in the papillary muscles, subendocardium, and myocardium showing partial ischemic changes. The grossly evident fibrotic lesion in the left ventricular wall was indicative of obsolescent ischemic change (Figures 3c and 4e).

The kidneys measured 174 and 170 g, with neither signs of atrophy nor significant discoloration. Histologically, very few brown granular casts were observed. However, herniation of the tubular epithelium into the glomerulus was noted, and findings indicated the effects of jaundice (Figure 4f).

Mild hemorrhagic changes were observed in the gastrointestinal mucosa as well as the urinary bladder, supporting the clinical diagnosis of coagulopathy, but no microthrombi were seen.

The spleen weighed 98 g and the lungs 202 g and 306 g, without significant findings.

Based on these findings, the cause of death was attributed to the thyroid storm, resulting in irreversible liver injury and HF.

## 4. Discussion

One of the major mechanisms proposed in the pathogenesis of ALF associated with thyrotoxicosis is relative hepatic ischemia. In thyrotoxicosis, the metabolic demand of hepatocytes markedly increases, whereas hepatic blood flow does not adequately increase, making the pericentral zone (Zone 3) vulnerable to ischemia [5]. In addition, excessive thyroid hormones can induce hepatocyte apoptosis via mitochondrial pathways [6]. Secondary hepatic congestion due to thyrotoxic heart failure and cholestatic liver injury caused by MMI [7] may also contribute.

In our case, the patient had no prior liver disease or imaging abnormalities before admission and was considered to have developed ALF. Autopsy revealed extensive hepatocyte necrosis, dropout, and marked congestion, and findings consistent with pathological features reported after thyroidectomy or liver transplantation for thyroid storm [8, 9]. These results suggest that relative hepatic ischemia and heart failure played a major role in the development of ALF. Similar findings of pericentral necrosis and bile duct proliferation have also been reported in autopsy cases of thyroid storm and in liver transplantation or thyroidectomy specimens [8, 9].

At autopsy, the liver weighed only 700 g, showing marked atrophy compared with previous reports (Table 1) [10]. This finding likely reflects multiple factors, not only delayed medical intervention but also relative hepatic ischemia under a hypermetabolic state, congestive hepatopathy secondary to thyrotoxic heart failure, and direct hepatotoxic effects of thyroid hormones. In addition, the patient experienced significant weight loss (approximately 5 kg within 10 days) and cachexia (BMI 17.7), suggesting that starvation‐related hepatocyte atrophy may also have contributed to the pathological process [11].

**Table 1:** Cases of thyroid storm with liver dysfunction. Extracted and modified from Reference [10].

**The year**	**Author**	**Journal name**	**Sex**	**Age**	**Liver weight (g)**	**Liver macroscopy**	**Liver histology**
1990	Oshima	The Japanese Journal of Legal Medicine	F	45	1000	Slightly stiff, finely granular cut surface with increased blood volume	Centrilobular necrosis, partial hemorrhage, fibrosis, regenerative nodule with central portal area, NRH
1992	Hosojima	Clinical Endocrinology	M	50	NA	NA	Centrilobular necrosis, congestion
1998	Kitamura	The Research and Practice in Forensic Medicine	F	34	750	Patchy pale yellow‐red and reddish‐brown colored	Sinusoidal dilatation, loss of hepatocytes with congestion
2006	Tatemichi	Kanzo	M	38	970	Atrophy	Centrilobular necrosis, few inflammatory cell infiltration
2006	Tatemichi	Kanzo	M	56	760	NA	Centrilobular necrosis and biliary congestion, few inflammatory cell infiltration
2010	Kuo	Journal of the Chinese Medical Association	M	63	NA	NA	Centrilobular hemorrhagic necrosis, hepatocyte loss, bile canaliculus proliferation, bile congestion
2015	Sakata	Hakodate Medical Journal	M	55	920	Finely granular cut surface	Hepatocyte loss, bile canaliculus proliferation, bile congestion
2025	Suzuki	Presently reported case	F	70	700	Atrophic with severe biliary stasis	Fibrotic enlargement of the portal area with pseudobile duct formation and lymphocytic inflammation. Severe congestion

Abbreviations: NA, not available; NRH, nodular regenerative hyperplasia.

Similar findings of pericentral necrosis and bile duct proliferation have been reported in autopsy cases of thyroid storm and in liver transplantation or thyroidectomy specimens [8, 9].

Recently, Yoshimura et al. reported that progression of coagulopathy and fibrosis was strongly associated with poor outcomes in patients with liver failure [12]. In our patient, PT activity was already reduced to 41.5%, and INR prolonged to 1.72 on admission and subsequently remained around 40% with INR worsening to 2.22 (Figure 2). These values fall within the poor prognosis group identified by Yoshimura et al., suggesting that our patient carried multiple unfavorable prognostic factors [10].

We also compared our case with a previously reported case in 2015, in which HF resulted from AF, with an initial ejection fraction (EF) of 20% upon admission. In our case, HF was also associated with AF, although EF upon admission was relatively high at 59%. Both our case and the 2015 case progressively worsened, in accordance with the fact that HF is considered a major cause of death in patients with thyroid storm. As to the cause of HF, one study suggests that autoimmune mechanisms may contribute to myocardial dysfunction in thyroid storm‐induced cardiomyopathy [13]. In another case, lymphocytic myocarditis was reported in an autopsy case of refractory congestive HF due to thyrotoxicosis [7]. However, the possibility of myocarditis or autoimmune mechanisms, including lymphocytic infiltration, was ruled out in our case. Additionally, age is a significant prognostic factor in thyroid storm. A study from Germany shows that case fatality was below 2% in persons aged < 60 years and markedly higher in older persons (males 17 times and females 8 times) [14]. Our patient was older, 70 years old, indicating she was at higher risk of fatality from a thyroid storm.

Liver transplantation is the optimal therapeutic option for ALF secondary to thyroid storm, but it is generally indicated for younger patients with preserved overall condition [8, 9, 15]. In the present case, transplantation was not performed due to advanced age (70 years, defined as old age ≥ 65 years) and the presence of multiorgan failure. Aging is associated with impaired regenerative potential and decreased tolerance to intensive therapeutic procedures, such as organ transplantation [16], thereby restricting its applicability. Although age alone is not an absolute contraindication, advanced age and poor general condition are recognized as relative contraindications for liver transplantation. In addition, the presence of multiorgan failure in our patient implied severe cardiopulmonary compromise and uncontrolled systemic condition, which are considered absolute contraindications for transplantation [17, 18].

In summary, this case suggested that multiple pathophysiological mechanisms were involved in the development of ALF associated with thyroid storm. In particular, relative hepatic ischemia, hepatic congestion, and the direct effects of thyroid hormones likely acted in combination to account for the clinical course and pathological findings. Moreover, the prominent contribution of heart failure to the deterioration of the clinical course was consistent with previous reports, reaffirming it as one of the major mechanisms in thyroid storm.

## 5. Conclusion

This case represents a rare autopsy report of ALF complicated by heart failure secondary to thyroid storm, leading to a fatal outcome. Pathologically, multifactorial hepatic injuries such as centrilobular necrosis, bile duct proliferation, and marked hepatic atrophy were observed, which were considered to result from the combined effects of relative hepatic ischemia, congestive hepatopathy, and the direct hepatotoxicity of thyroid hormones. Furthermore, heart failure contributed substantially to the deterioration of the clinical course, consistent with previous reports, and reaffirmed its role as a major cause of death in patients with thyroid storm. In this case, the degree of hepatic atrophy was particularly severe, potentially reflecting disease progression and poor prognostic factors. While successful liver transplantation has been reported in younger patients with preserved general condition, its indication in elderly patients remains challenging and requires careful consideration. Therefore, early recognition and multidisciplinary management before the development of irreversible liver injury and heart failure are of critical importance.

**Nomenclature**
LFTs: liver function tests
ALF: acute liver failure
JTA: Japan Thyroid Association
bil: bilirubin
HF: heart failure
AF: atrial fibrillation
BWPS: Burch–Wartofsky Point Scale
MMI: methimazole
KI: potassium iodide
PT%: prothrombin time activity percentage
CHDF: continuous hemodialysis and filtration
EF: ejection fraction
AST: aspartate transaminase
ALT: alanine aminotransferase

## Ethics Statement

Ethical approval was not required for this case report.

## Consent

Written informed consent was obtained from the patient (or the patient’s guardian) for the publication of this case report and any accompanying images.

## Disclosure

All relevant sources have been cited appropriately in the manuscript.

## Conflicts of Interest

The authors declare no conflicts of interest.

## Author Contributions

S.M. and Y.K. helped to analyze the pathological data and provided the macroscopic and microscopic images.

## Funding

No funding was received for this manuscript.

## Data Availability

The data that support the findings of this study are available on request from the corresponding author. The data are not publicly available due to privacy or ethical restrictions.
